# Developmental temperature induces consistent patterns of plasticity in lower, but not upper, critical thermal limits in threespine stickleback (*Gasterosteus aculeatus*)

**DOI:** 10.1038/s41598-026-73463-1

**Published:** 2026-10-08

**Authors:** Brenna C.M. Stanford, Sara J. Smith, Sean M. Rogers

**Affiliations:** 1 Department of Biological Sciences, University of Calgary, Calgary, AB Canada; 2 Bamfield Marine Sciences Centre, Bamfield, BC Canada; 3 Biology, Mount Royal University, Calgary, AB Canada

**Keywords:** Developmental biology, Ecology, Ecology, Neuroscience, Zoology

## Abstract

Developmental plasticity, where early-life environmental cues shape developmental trajectories with lasting impact into adulthood, may play an important role in determining how species respond to changing thermal environments. We used marine threespine stickleback (*Gasterosteus aculeatus*) to investigate whether temperature experienced during embryonic development has persistent effects on critical thermal limits as adults. Egg clutches from six families of pure marine F2s were split across three developmental temperatures (12 °C, 15.5 °C, and 22 °C), transitioned to 15.5 °C once hatched, and reared to adulthood under common-garden conditions, before being assayed for critical thermal minimum (CT_min_) and maximum (CT_max_). Developmental temperature was found to have significant, persistent effects on CT_min_, where the direction of change tracked rearing temperature (i.e., CT_min_ decreased as early life temperature decreased). While the extent of change (i.e., slope of reaction norm) and inherent values (i.e., height of reaction norm) varied across families, all families shared the same trend. In contrast, the effect of developmental temperature on CT_max_ was minimal, with inconsistent patterns across families. The ability of marine stickleback to lower their CT_min_ through developmental plasticity may help maintain phenotype-environment match under changing conditions; however, this will depend on congruence between early life and adult environments.

## Introduction

Temperature plays a substantial role in generating and structuring patterns of global biodiversity^1–3^. Adaptation across evolutionary history has shaped species’ thermal tolerance and preference, contributing to the thermal niches that they now occupy^2,4–7^. However, increasing average temperatures, as well as fluctuations in temperature extremes associated with climate change, are exposing species to temperatures outside of their historic norms^8,9^. With the intensity of these observed environmental changes only projected to increase^10,11^, it is critical to determine the mechanisms by which populations and species tolerate and adapt to thermal stresses^12–14^.

Phenotypic plasticity, the ability of a genotype to give rise to different phenotypes depending on environmental cues, is one mechanism by which organisms can rapidly adjust temperature-associated phenotypes in response to their environment^15,16^. Thermally cued phenotypic plasticity has been observed in a broad range of traits across species, including physiological processes such as metabolic rates^17,18^, behavioural traits such as thermoregulatory behaviour^19^, and life-history traits such as reproductive timing^20,21^. Ectothermic organisms, whose body temperatures are largely dictated by environmental temperature, are particularly sensitive to temperature changes and extremes^7,22,23^. Thermal limits in ectothermic species are complex traits which are set by cellular, physiological, and behavioural inputs, and vary across species, sex, genetic background, and evolutionary history^4,10,22,24,25^. In response to shifting thermal environments, ectothermic species have evolved mechanisms to remain within their zone of tolerance^19^. Ectotherms have even been found to be able to adjust their thermal limits in response to thermal acclimation^26^ through phenotypic flexibility, a form of phenotypic plasticity distinguished by reversible changes in phenotype^27^.

A less investigated form of plasticity is developmental plasticity, where environmental cues experienced early in life can result in different developmental trajectories, with potentially profound impacts on phenotype-environment match throughout the lifetime of an organism^28,29^. If the early-life environment is reflective of the adult environment, then this may ‘prime’ individuals, resulting in a fitness advantage^30–32^. However, developmental conditions may also constrain proper development, generating less fit phenotypes, or the environment may change between early-life development and adulthood, resulting in phenotype-environment mismatch^31^. Notably, there is evidence that developmental plasticity may shape thermal limits, though observations have differed between lower and upper thermal limits^33^. This may be due, in part, to different ‘physiological boundaries’ for upper and lower critical thermal tolerance, where the upper thermal limit of many species is likely already close to their intrinsic limit^4,34^. While developmental temperature has been observed to have long-term impacts on various phenotypes throughout the lifetime of ectotherms^35,36^, its influence on shaping thermal tolerance remains poorly understood^28^.

Threespine stickleback (*Gasterosteus aculeatus*) is a species that is well-suited for testing hypotheses of plasticity and adaption. Following post-glacial range expansion, marine threespine stickleback exhibited widespread colonization of freshwater and brackish habitats across the Holartic region^37,38^, highlighting their ability to tolerate and adapt to novel environmental conditions such as parasites^39^, salinity^40^, and temperature^26,34,40,41^. Current marine threespine stickleback populations are largely considered ‘contemporary ancestors’ (but see^42^ when compared to their derived freshwater counterparts and can be used to give insight on the characteristics that allowed for such widespread distribution^43^. They have also continued to adapt to marine environments^42^, which include variable thermal regimes^44^. Marine stickleback have been shown to be able to decrease their lower thermal limits through rapid evolution^34^, as well as through phenotypic flexibility as adults^26^, and increase CT_max_ under simulated heat wave conditions^45^. While egg temperature has been observed to have lasting impact on adult phenotypes, including on gene expression and regulation^46,47^, reproductive investment^48,49^, and body shape^50^, it remains unknown if thermal limits exhibit developmental plasticity.

Here, we investigated the impact of developmental plasticity on critical thermal limits. We exposed marine threespine stickleback eggs to three distinct temperatures during development, then reared the hatched juveniles to adulthood under common-garden conditions before assessing their upper and lower critical thermal tolerance. Our objectives were to (i) determine if CT_min_ and/or CT_max_ exhibit plasticity inducible by early developmental cues, (ii) assess if the directionality of plasticity in CT_min_ and CT_max_ mirrors the developmental temperature treatment (i.e., decreased CT_min_ and CT_max_ if developed at a cold temperature), and (iii) examine whether CT_min_ and CT_max_ show similar levels of plasticity.

## Methods

### Wild stickleback collection and F1 crosses

Over 90 wild marine threespine stickleback were collected from Bamfield Inlet, Bamfield, Vancouver Island BC, Canada (48°49’01.4"N 125°09’11.0"W) in June 2019 and held in flow-through seawater tanks at the Bamfield Marine Science Centre (BMSC), Bamfield, Vancouver Island BC. Four F1 families were generated in August 2019, and eggs were developed in closed tanks of “brackish” salinity at 20 parts per thousand (ppt) for 14 days before fresh marine water was added from the Bamfield Inlet (approximately 30 ppt). Beginning at one-week post-hatch, juveniles were fed live *Artemia* nauplii to satiation twice a day until they reached a length of approximately 2 cm, before transitioning to Hikari bloodworms. In late February 2020, each F1 family was tagged with a distinctive visible implant elastomer tag (Northwest Marine Technology, Inc.) and combined into a large tank (*N* = 72) with the intention of rearing them to adulthood at this location. In response to COVID-19 restrictions, and to ensure the animals could be appropriately monitored, we transported all fish to the Life and Environmental Sciences Animal Resource Centre (LESARC) at University of Calgary, Calgary, Alberta, Canada in early June 2020. Fish were reared to adulthood in 30-gallon closed tanks (families randomly spread across tanks in the same conditions) at 12–14 °C and 30 ppt (achieved with Instant Ocean^®^ Sea Salt).

### F2 crosses and experimental treatments

**Fig. 1:**
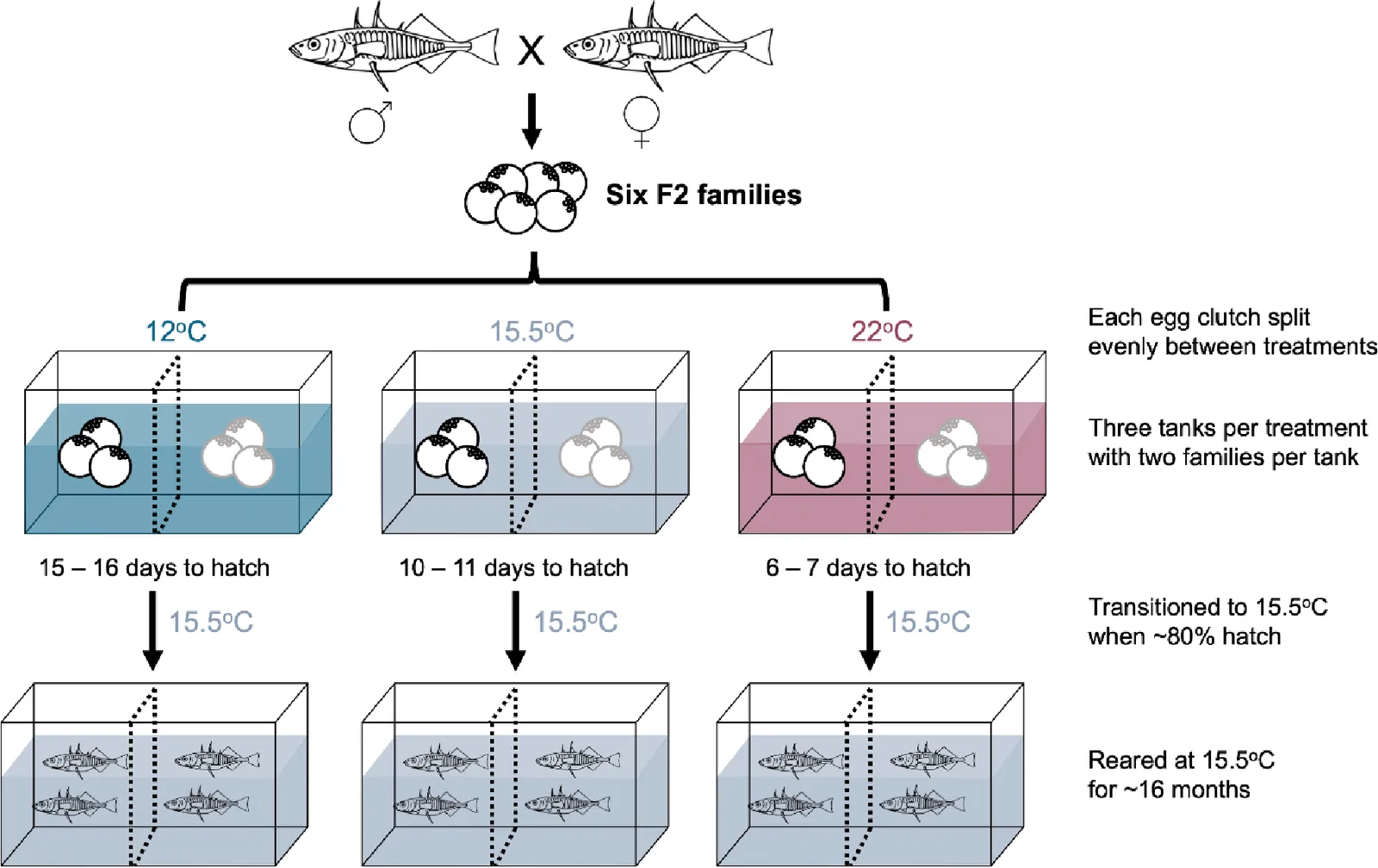
Experimental design. Six families of F2 marine threespine sticklebacks were generated and egg clutches were split between three developmental temperatures of 12 °C, 15.5 °C, and 22 °C with two families per tank (*n* = 3 tanks per temperature). Once ~ 80% of eggs had hatched the 12 °C and 22 °C tanks were transitioned to 15.5 °C over about 12 h. At age 12 and 13 months, fish were assayed through thermal trials to determine their maximum and minimum thermal limits.

Independent F2 families were generated by crossing a male of one F1 family with a female of a different F1 family to create six families in mid- to late October 2020. Clutch size was determined by counting total number of eggs with successful fertilization (i.e., blastodisc formation after about 20 min) and were split evenly across divided 30-gallon tanks (30 to 40 eggs per family per treatment) at 20 ppt and held at three rearing temperatures of 12 °C, 15.5 °C, and 22 °C using JBJⒸ Arctica 1/10 hp titanium aquarium chillers and Fluval^®^ E50 electronic heaters (Fig. 1). These temperatures were selected based on previous experiments that confirmed these are near lower and upper limits for development without mortality for marine threespine stickleback^47,50,51^. Two families were reared in each tank and there were three replicate tanks for each treatment for a total of nine tanks (three at each rearing temperature; Fig. 1). Eggs were checked daily until hatch and nonviable eggs were removed and recorded. Once roughly 80% of the eggs had hatched, the 12 °C and 22 °C tanks were brought to 15.5 °C over 12 h (Fig. 1). Any unhatched eggs were removed and recorded. F2 fish were fed following the same regime as above and were reared under these conditions until their experimental endpoint as adults (Fig. 1). Once the F2 fish reached adulthood, they were given unique elastomer tags for identification so that individuals could be tracked throughout the experimental trials.

### Body size and weight measurements

Body weight (M; 0.01 g) (Ohaus^®^ Scout^®^ SPX222) and standard length (SL; 0.1 cm) were measured before CT_min_ and CT_max_ trials (at approximately 12 and 13 months post-hatch, respectively). These values were used to calculate Fulton’s body condition (K), as previous studies have found correlation between this proxy of fitness and thermal tolerance in other fish species^25,52^.

### Critical thermal trials

To determine the lower and upper thermal limits, critical minimum (CT_min_) and maximum (CT_max_) thermal trials were conducted following methods outlined in^34] and [10^ on the fish at one-year post-hatch. An average of 8–11 fish per family per developmental treatment were assayed for CT_min_ and CT_max_ (*N* = 177 fish; n_12°C_=63, n_15.5 °C_=57, n_22°C_=57). All trials were performed within approximately 40 days, with all fish undergoing the CT_max_ trial first, recovering for at least 21 days, and then undergoing the CT_min_ trial. To conduct the trials, 1000 mL glass beakers were filled with 20 ppt water and placed across two experimental tanks. Each beaker was aerated with an air stone and contained a digital thermometer (Cooper-Atkins DFP450W-0-8 Waterproof Reduced-Tip). Fish were fasted for 24 h and acclimated in an individual beaker for 15 min before the trial started. Each trial started at approximately 15.5 °C and then the temperature was decreased or increased at a rate of approximately 0.33 °C per minute. To do so, water was circulated from a header tank through the experimental tanks that was chilled using a combination of JBJⒸ Arctica 1/10 hp titanium aquarium chillers, ice, and dry ice to test CT_min_, or heated using a combination of Fluval^®^ E50 electronic heaters and boiled water to test CT_max_. Once a fish reached their sub-lethal thermal limit, characterized by a loss of equilibrium^53^, they were removed from the beaker and allowed to recover in a holding tank at 15.5 °C before being returned to their experimental tank. No mortalities occurred during the trials.

### Sex determination

At experimental endpoint, fish were euthanized using an overdose of clove oil and visually sexed during dissection. Fin clips were taken for each fish, stored in 95% EtOH, and subset of samples (approximately 30%) were genetically sexed to confirm visual results. To do so, DNA was isolated using a modified HOTSHOT protocol^54,55^ and amplified at the *Idh* locus via polymerase chain reaction (PCR)^56^ with 100% agreement between visual and genetic sex assignment.

### Statistical analysis

All statistical analyses were completed using R v.4.6.0^57^ in R Studio^58^. One-way ANOVAs were used to test if developmental temperature had a significant influence on days to hatch and percent of fertilized eggs that survived to hatch. Thermal tolerance data was filtered to only contain fish that had experienced both thermal trials. For each fish, Fulton’s body condition ($\:K=\:\frac{M}{{SL}^{3}}$ ) at time of CT_min_ and CT_max_ trial was calculated.

Initial evaluation of distribution of response variables (CT_min_ and CT_max_) was done using the *descdist* and *fitDist* functions of the **fitdistrplus** v.1.1–11^59^. All response variables followed non-normal distributions so *fitDist* was run^60,61^ and the suitability of the best fit distributions was assessed using *histDist* from the **gamlss** v.5.4–20 package. Critical thermal tolerance values (CT_min_ or CT_max_) were fit with two complementary models, with both models for CT_min_ best fit to the ex-Gaussian distribution, and CT_max_ best fit to the Weibull distribution. First, to determine if developmental temperature induced significant shifts (i.e., developmental plasticity) in CT_min_ or CT_max_ at the population level, critical thermal tolerance values were fit with Model 1 (*CT ~ treatment + sex + body condition + random(family)*), where body condition was at the time of trial, and family was included as a random effect to account for the non-independence of siblings. To further investigate whether patterns of plasticity were shared across genetic backgrounds, critical thermal tolerance values were fit with Model 2 (*CT ~ treatment × family + sex + body condition*), where family was treated as a fixed effect to test the effects of developmental temperature within family i.e., as a proxy for same genetic background.

Residuals of all final models were visualized, with no models violating assumptions. For Model 2, pairwise post-hoc contrasts with a Benjamini-Hochberg correction were conducted within family using *emmeans* from the **emmeans** package v.1.10.0^62^.

## Results

### Days to hatch and percent survival

Developmental temperature impacts days to hatch, with eggs developed at 12 °C requiring 15–16 days, 15.5 °C requiring 10–11 days, and 22 °C requiring 6–7 days (one-way ANOVA, *F*(2, 6) = 183, *p* < 0.05). There was no significant difference in percent of eggs hatched between the temperature treatments, with 93.9 ± 6.9% hatch at 12 °C, 92.2 ± 4.0% hatch at 15.5 °C and 86.9 ± 7.8% hatch at 22 °C (mean ± SD; one-way ANOVA, *F*(2, 15) = 1.93, *p* > 0.05).

### General patterns of CT_min_ and CT_max_

**Fig. 2:**
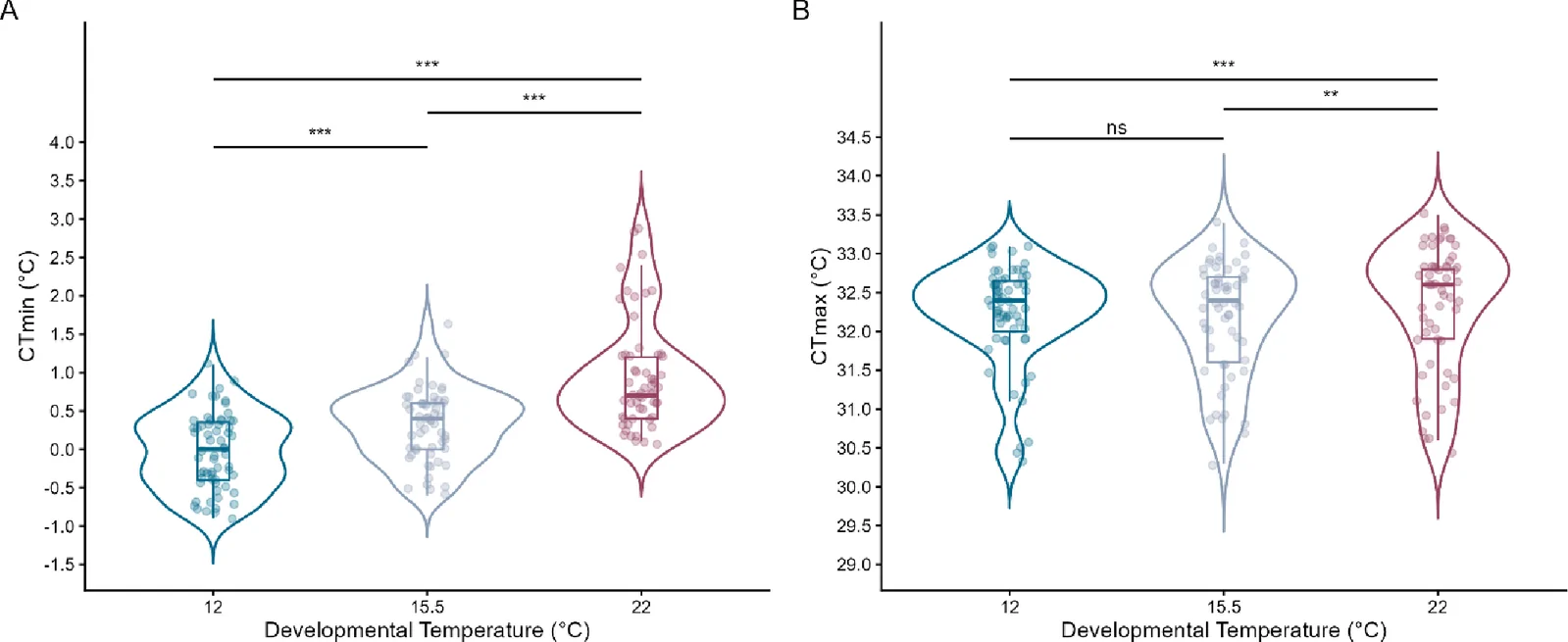
(**A**) Critical minimum (CT_min_) and (**B**) Critical maximum (CT_max_) at each developmental temperature treatment (12 °C, 15.5 °C, and 22 °C) for all families plotted with the median bounded by the 25th and 75th quartiles and whiskers that extend to the furthest point or 1.5 times the inter-quartile range. Contrasts in CT_min_ and CT_max_ between fish developed at 12 °C, 15.5 °C, and 22 °C are shown with the black lines, where significance thresholds are indicated as *** *p* < 0.001, ** *p* < 0.01, * *p* < 0.05, ns not significantly different.

**Fig. 3:**
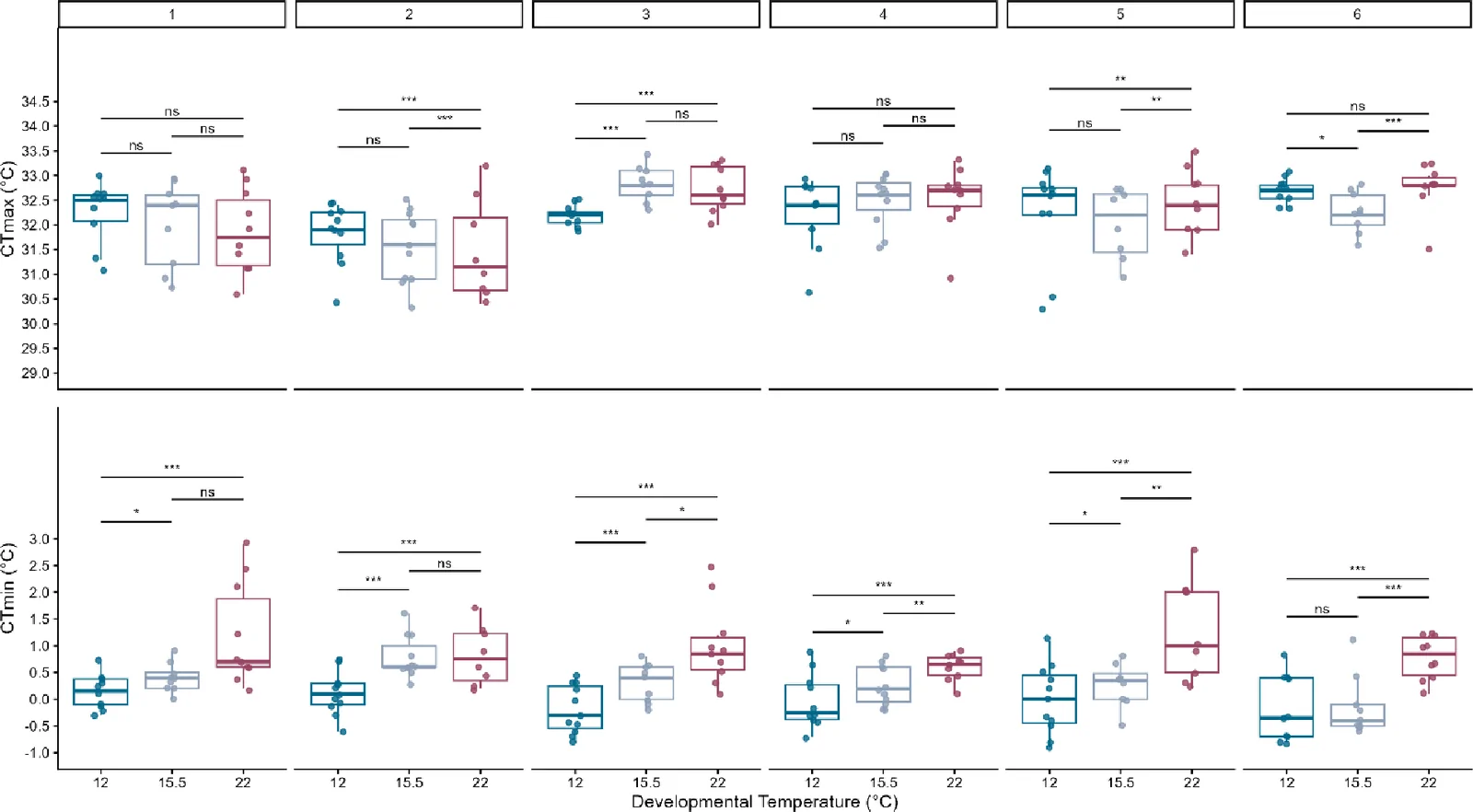
Critical minimum (CT_min_) and maximum (CT_max_) at each developmental temperature treatment (12 °C, 15.5 °C, and 22 °C) within each family (*N* = 6) plotted with the median bounded by the 25th and 75th quartiles and whiskers that extend to the furthest point or 1.5 times the inter-quartile range. Benjamini-Hochberg corrected contrasts in CT_min_ and CT_max_ between fish developed at 12 °C, 15.5 °C, and 22 °C within each family are shown with the black lines, where significance thresholds are indicated as *** *p* < 0.001, ** *p* < 0.01, * *p* < 0.05, and ns is not significantly different.

Upper and lower thermal tolerance was found to vary between families with the range of values shifting across developmental temperatures (Table 1; Figs. 2A and B and 3). For fish developed at 12 °C, there was a range of 0.33 °C in average CT_min_ (-0.19 ± 0.44 °C in family 3 to 0.14 ± 0.32 °C in family 1) and 0.86 °C in average CT_max_ (31.82 ± 0.61 °C in family 2 to 32.68 ± 0.27 °C in family 6) between families (Table 1; Fig. 3). For fish developed at 15.5 °C, there was a range of 0.92 °C in average CT_min_ (-0.14 ± 0.56 °C in family 6 to 0.78 ± 0.39 °C in family 2) and 1.28 °C in average CT_max_ (31.54 ± 0.73 °C in family 2 to 32.82 ± 0.35 °C in family 3) between families (Table 1; Fig. 3). For fish developed at 22 °C, there was a range of 0.90 °C in average CT_min_ (0.60 ± 0.24 °C in family 6 to 1.30 ± 0.92 °C in family 4) and 1.27 °C in average CT_max_ (31.48 ± 0.48 °C in family 2 to 32.75 ± 0.15 °C in family 4) between families (Table 1; Fig. 3).

**Table 1:** Proportion of females and males, total number of samples, and mean ± SD for CT_min_ and CT_max_ (calculated combining females and males) per developmental temperature per family.

Family	Developmental Temperature	Num. of Females	Num. of Males	Total Sample Size	Average CT_min_ ± SD (˚C)	Average CT_max_ ± SD (˚C)
1	12˚C	4	6	10	0.14 ± 0.32	32.25 ± 0.61
	15.5˚C	5	4	9	0.40 ± 0.27	31.99 ± 0.86
	22˚C	5	5	10	1.18 ± 0.94	31.85 ± 0.84
2	12˚C	6	5	11	0.11 ± 0.39	31.82 ± 0.61
	15.5˚C	7	4	11	0.78 ± 0.39	31.54 ± 0.73
	22˚C	3	5	8	0.81 ± 0.55	31.48 ± 1.02
3	12˚C	8	3	11	-0.19 ± 0.44	32.18 ± 0.20
	15.5˚C	3	6	9	0.30 ± 0.36	32.82 ± 0.35
	22˚C	5	5	10	1.01 ± 0.76	32.72 ± 0.45
4	12˚C	4	6	10	-0.03 ± 0.51	32.24 ± 0.72
	15.5˚C	7	4	11	0.25 ± 0.37	32.48 ± 0.53
	22˚C	3	7	10	0.60 ± 0.24	32.53 ± 0.67
5	12˚C	5	6	11	-0.01 ± 0.63	32.25 ± 0.96
	15.5˚C	2	6	8	0.26 ± 0.42	32.01 ± 0.71
	22˚C	4	5	9	1.30 ± 0.92	32.47 ± 0.67
6	12˚C	5	5	10	-0.17 ± 0.61	32.68 ± 0.27
	15.5˚C	4	5	9	-0.14 ± 0.56	32.24 ± 0.41
	22˚C	3	7	10	0.77 ± 0.41	32.75 ± 0.48

### Plasticity in CT_min_

Developmental temperature shifted the range of values in lower thermal tolerance in the direction of the thermal cue (Model 1: 12 °C vs. 15.5 °C: *t*= 4.778, *p*< 0.0001; 15.5 °C vs. 22 °C: *t*= 5.788, *p*< 0.0001; 22 °C vs. 12 °C: *t*= 10.768, *p*< 0.0001) (Fig. 2A). Fish developed at 22 °C had CT_min_ values that ranged from 0.1 °C to 2.9 °C, with a mean of 0.94 ± 0.70 °C and fish developed at 12 °C ranged from − 0.9 °C to 1.1 °C with a mean of −0.025 ± 0.49 °C (Fig. 2A). Fish developed at 15.5 °C had an intermediate range of CT_min_ values that spanned from − 0.6 °C to 1.6 °C with a mean of 0.323 ± 0.48 °C (Fig. 2A). This shift was reflected in the proportion of individuals with CT_min_ below 0 °C, which decreased from 49% in fish developed at 12 °C to 23% in fish developed at 15.5 °C and 0% in fish developed at 22 °C (Fig. 2A). CT_min_ was not found to be associated with body condition (*t* = -1.386, *p* = 0.168). Males were found to have a slightly higher CT_min_ than females (*t* = 2.310, *p* = 0.0221).

Comparing across families (Model 2), developmental temperature was observed to induce consistent plastic changes in CT_min_, where fish developed at 12 °C had lower CT_min_ than those developed at 22 °C in all families (family 1: z = −4.070, *p* = 0.0001; family 2: z = −3.789, *p* = 0.0003; family 3: z = −5.762, *p* < 0.0001; family 4: z = −5.085, *p* < 0.0001; family 5: z = −6.620, *p* < 0.0001; family 6: z = −7.097, *p* < 0.0001) (Fig. 3). These effects of experiencing 12–22 °C throughout development on adult CT_min_ ranged from a minimal change of 0.63 °C (family 4: −0.030 ± 0.51 °C at 12 °C to 0.60 ± 0.24 °C at 22 °C) to a maximal change of 1.31 °C (family 5: −0.0091 ± 0.63 °C at 12 °C to 1.30 ± 0.92 °C at 22 °C) (mean ± SD) (Table 1; Fig. 3). The CT_min_ of fish developed at 15.5 °C was never below that of 12 °C fish or higher than that of 22 °C (Fig. 3), however, while consistent differences between fish developed at 12–22 °C were observed, how these values shifted in comparison to fish developed at 15.5 °C varied between families (Fig. 3).

### Plasticity in CT_max_

Unlike CT_min_, developmental temperature was found to only weakly shift the observed range of values for CT_max,_ where the lowest CT_max_ was 30.3 °C, 30.3 °C, and 30.4 °C, and the highest CT_max_ was 33.1 °C, 33.4 °C, and 33.5 °C for fish developed at 12 °C, 15.5 °C, and 22 °C, respectively (Fig. 2B). The proportion of individuals at the higher range of CT _max_ values was seen to increase corresponding with developmental temperature, however, this resulted in only a 0.1 °C increase in mean CT _max_, with mean values of 32.2 ± 0.08 °C, 32.2 ± 0.10 °C, and 32.3 ± 0.11 °C for fish developed at 12 °C, 15.5 °C, and 22 °C, respectively (12 °C vs. 15.5 °C: *t* = 0.437, *p* = 0.663; 15.5 °C vs. 22 °C: *t* = 3.314, *p* = 0.001; 22 °C vs. 12 °C: *t* = 3.905, *p* < 0.0001) (Fig. 2B). Body condition was not found to be associated with CT_max_ (*t =* 1.611, *p* = 0.109). Sex was found to be associated with CT_max_, with males and females having mean values of 32.4 ± 0.68 °C and 32.1 ± 0.74 °C, respectively (*t* = -5.648, *p* < 0.0001).

The effects of developmental temperature on CT_max_ were found to be inconsistent across families (Fig. 3). When comparing CT_max_ of fish developed at 22 °C compared to 12 °C, no change in CT_max_ was observed for family 1 (z = -0.116, *p* = 0.908), family 4 (z = -1.356, *p* = 0.316), or family 6 (z = -2.094, *p* = 0.073) (Fig. 3). Differences were observed between 22 °C and 12 °C for family 2 (z = -4.009, *p* = 0.0002), family 3 (z = -5.393, *p* < 0.0001), and family 5 (z = -3.110, *p* = 0.0048), however, the direction of change in mean CT_max_ values between these treatments was variable (Fig. 3). When compared to fish developed at 15.5 °C, development at 12 or 22 °C had either no effect on CT_max_ (family 1 and 4), an effect only at 12 °C (family 3) or 22 °C (family 2 and 5), or an effect of both 12 °C and 22 °C (family 6) (Fig. 3).

## Discussion

In this study, we investigated the impact of developmental plasticity on critical thermal tolerance in adulthood using controlled crosses of marine threespine stickleback. We found that developmental temperature (i.e., temperature experienced at the egg stage) significantly impacted days to hatch, but not survival. Developmental temperature also had persistent effects on CT_min_, influencing patterns more than a year later in the direction of the developmental temperature (e.g., decreased CT_min_ from cooler developmental temperatures), where patterns of plasticity were shared across all families tested. On the other hand, the effect of developmental temperature on CT_max_ was minimal, with inconsistent effects across families. Collectively these results indicate that thermal developmental plasticity plays a role in setting lower thermal tolerance, while its effect on upper thermal tolerance in marine threespine stickleback appears minimal and family-specific.

The differences in the developmental plasticity between CT_max_ and CT_min_ found here are consistent with studies in other ectotherms. Thermal cues experienced early in life have previously been shown to result in little to no change in CT_max_^33,63–65^. Aspects of CT_min_ and CT_max_ have been correlated, such as in inherent values^10^ and phenotypic flexibility^66^, however, the divergent patterns observed here, and in previous research, indicate that developmental plasticity within these traits may be shaped by interactions between behaviour and physiology^12,67^. Marine threespine stickleback have phenotypic flexibility in both CT_min_ and CT_max_ and can alter these values by several degrees in response to thermal acclimatation^26,45^. However, the timing of physiological remodelling may differ between cold versus heat stresses (e.g., slower acclimation response in oxidative muscle in response to cold versus warm conditions in *Oncorhynchus mykiss*^68^). As well, ectotherms have thermoregulatory behavioural mechanisms available to either fully or partially compensate for the effects of thermal stress, and they can use such mechanisms to remain within a zone of tolerance (below acute stress) for short windows of time or until acclimatization occurs^69^. However, thermoregulatory behavioural mechanisms, such as selection of time of day to be active and use of environmental heterogeneity^12,70,71^, have less capacity to compensate for cold body temperature compared to warm^66^. Additionally, high temperatures induce frantic movements within most fishes such that they are more likely to move away from the thermal stressors, while colder temperatures decrease movement, potentially further decreasing ability to behaviourally thermoregulate^72,73^. These factors can negatively impact the ability of fishes to escape cold stress, as well as increase the time exposed to cold stress, potentially favouring mechanisms that can ‘prime’ individuals for these conditions^30–32^. In contrast, marine threespine stickleback may maintain variability in CT_max_ between individuals, instead of predicting that one phenotype will be advantageous over the other into adulthood^74,75^, where individuals with initially low inherent CT_max_ can rapidly increase to values near physiological limits via thermal acclimation^45^. Such inferences emphasize the importance of concurrently examining both low and high thermal stressors in fishes to understand potential ecological impacts of thermal tolerance.

It is also possible that the patterns found here are the knock-on effects of processes modified to ensure proper development across temperatures, rather than arising by selection acting on the phenotypes at the adult stage^31,35,76,77^. Temperature is seen to impact nearly all developmental processes, especially in ectotherms^35,36^, and yet, successful development can occur over a range of temperatures^76^. For example, we found that while developmental temperature spanning 12 °C to 22 °C significantly altered time to hatch, it did not impact survival, which aligns with previous research in marine threespine stickleback^46^. This shortened developmental period under warmer conditions is largely the consequence of increased metabolic rate in ectothermic species^36,76^. To compensate, embryos may alter pathways underlying metabolic processes (e.g., through epigenetic reprogramming^78^, which may have lasting impacts on factors influencing thermal tolerance such as metabolic rate^79^ and lipid composition^80^. Regardless of the processes driving developmental plasticity within thermal tolerance, thermal conditions experienced in early life expose phenotypic variation, especially for CT_min_, that may be acted on by selection under future conditions^74^.

Since thermal phenotypic plasticity may improve fitness under thermal stress, it may also play a key role in responses to climate change^15,81^. Studies on ectothermic responses to climate change have mostly focused on the effects of heat stress and have largely ignored cold stress^82^, including those on developmental plasticity and thermal tolerance^28,63–65^. However, it is the evolution of CT_min_ and not CT_max_ that has been shown to inform marine threespine stickleback range distributions and evolution under climate projections^10^. This is likely due to the fact that adult marine threespine stickleback already have upper critical thermal limits higher than what they are projected to experience under future climate change models for the west coast of North America^10^. However, increased environmental variability and extremes caused by climate change^8,9^, as well as range shifts northwards, may expose species to colder minimum temperatures than previously experienced^10,83^. As such, lower thermal tolerance is an important consideration when characterizing different species’ response to climate change^10,82,84^.

The ability of early-life temperature cues to shape adult phenotypes is highly dependent on conditions not exceeding the thermal limits of eggs, which for many marine ectotherms, have a smaller window of tolerance than adults^85,86^. For marine threespine stickleback, though adult CT_max_ is above the temperatures forecasted in climate projections^10^, egg and juvenile stages may still be vulnerable to the effect of high or low temperatures. Though embryos were seen here to be well tolerant of development at 22 °C, with other studies finding similar patterns up to 24 °C^46^, hard limits may exist around 25 °C^87^. While characterizing adult phenotypes is necessary to understand how they may tolerate environmental change, it requires embryos and juveniles to have sufficient plasticity to survive to adulthood^88^. Improved understanding of the capacity of plasticity to buffer thermal stress at each life-stage is necessary to predict how species may react to climate change^86,89^.

Through developmental plasticity, marine threespine stickleback were found to be able to shift CT_min_ by approximately 1 °C into adulthood. This trend was driven by a shift in the range of CTmin values; specifically, no individuals developed at 22 °C had a CT_min_ below 0 °C while those developed at 12 °C exhibited CT_min_ limits as low as -0.9 °C, with approximately 50% of individuals exhibiting a CT_min_ below 0 °C. Depending on environmental conditions, this phenotypic variation may result in individuals with varying fitness upon which selection can act^29,90^, which may contribute to the rapid evolutionary rate of lower CT_min_ observed in marine threespine stickleback^34^. As adult marine threespine stickleback in certain populations may already experience temperatures close to 0 °C^87^, the ability to adjust lower thermal limits through developmental plasticity and phenotypic flexibility^26^ may play a role in response to future conditions under climate change, including extreme weather events. To be beneficial though, the cues need to produce a phenotype that matches the environmental conditions. Marine threespine stickleback eggs are laid in eelgrass beds^91^ that occupy shallow, intertidal estuarine areas where the effects of climate change may be amplified^92,93^. If the thermal conditions experienced in early life are not reflective of the environment experienced by adults this may lead to increased phenotype-environment mismatch (e.g., warmer water in eelgrass beds cuing a higher CT_min_ when increased cold stress will be experienced at the adult stage^10,83^^32,94^. Ultimately, developmental plasticity is just one of the many factors, such as photoperiod and acclimation temperature^26^, that may shape CT_min_ across an individual’s life. Additionally, CT_min_ and CT_max_ are only two traits that contribute to thermal tolerance. Overall, future studies will benefit from investigating the impact of developmental plasticity on other thermally relevant traits under different ecological conditions to better understand how species will respond to climate change.

In this study, we showed that the temperatures marine threespine stickleback experience in early life can have persistent effects on setting critical thermal limits, particularly in CT_min_. While determining critical thermal limits, and the processes that shape them, are valuable contributions towards understanding a species’ response to environmental change^10,95^, it is important to remember that these values represent an organism’s absolute physiological limits in response to acute thermal stress. These limits are set by the complex interaction of internal and external factors (e.g., gene regulation throughout development^74^, genetic background^10^, and environmental conditions^25^ and there may be no single ‘best’ phenotype when considered in the context of other traits that contribute towards response to temperature^75^. Even if thermal developmental plasticity is found to not alter critical thermal limits, it may have substantial effects on other traits^96^, such as swim performance^97^, stress responses^98^, and thermal acclimation capacity^64^. Ultimately, further research that characterizes individual responses across multiple phenotypes^74,75^ is necessary to better understand how species’ have evolved to tolerate environmental variability and how these mechanisms may impact response to future conditions.

### Animal care and ethics research statement

All procedures were approved by the University of Calgary Animal Care Committee under protocol AC17-0138. Experiments were performed in accordance with all relevant institutional and national Canadian Council for Animal Care guidelines and regulations for the care and use of animals in research. Permission and collection permits were obtained, including a heritage investigation permit to conduct research in the territories of the Huu-ay-aht First Nations (HFN 2020-003), licenses from the Department of Fisheries and Oceans (DFO) to capture wild threespine stickleback (XR110 2020; XR134 202), an introduction and transfer certificates (ITC) to move these fish to the Bamfield Marine Sciences Centre and to the University of Calgary (ITC 124714; ITC 121300), and a domestic transfer permit from the Canadian Food Inspection Agency (CFIA D2020-106825). The University of Calgary held a Research Licence (#20–0108) from Alberta Environment and Parks Fish and Wildlife to maintain fish in contained facilities at the Life and Environmental Science Animal Research Lab, in compliance with the containment standards of the Canadian Food Inspection Agency (CFIA AQ 2020-0001-4). All permits are available upon request.

## Data Availability

All data and code are accessible from the corresponding author and will be published in Dryad or equivalent data repository.
